# Ataxia in Patients With Bi-Allelic *NFASC* Mutations and Absence of Full-Length NF186

**DOI:** 10.3389/fgene.2019.00896

**Published:** 2019-09-24

**Authors:** Malin Kvarnung, Mansoureh Shahsavani, Fulya Taylan, Mohsen Moslem, Nicole Breeuwsma, Loora Laan, Jens Schuster, Zhe Jin, Daniel Nilsson, Agne Lieden, Britt-Marie Anderlid, Magnus Nordenskjöld, Elisabeth Syk Lundberg, Bryndis Birnir, Niklas Dahl, Ann Nordgren, Anna Lindstrand, Anna Falk

**Affiliations:** ^1^Department of Clinical Genetics, Karolinska University Hospital, Stockholm, Sweden; ^2^Department of Molecular Medicine and Surgery, Center for Molecular Medicine, Karolinska Institutet, Stockholm, Sweden; ^3^Department of Neuroscience, Karolinska Institutet, Biomedicum, Stockholm, Sweden; ^4^Department of Immunology, Genetics, and Pathology, Science for Life Laboratory, Biomedical Centre, Uppsala University, Uppsala, Sweden; ^5^Department of Neuroscience, Biomedical Centre, Uppsala University, Uppsala, Sweden

**Keywords:** neurofascin, neuronal isoform *NF186*, ataxia, patient-specific induced pluripotent stem cells, neuroepithelial stem cells, neurites

## Abstract

The etiology of hereditary ataxia syndromes is heterogeneous, and the mechanisms underlying these disorders are often unknown. Here, we utilized exome sequencing in two siblings with progressive ataxia and muscular weakness and identified a novel homozygous splice mutation (c.3020-1G > A) in neurofascin (*NFASC*). In RNA extracted from fibroblasts, we showed that the mutation resulted in inframe skipping of exon 26, with a deprived expression of the full-length transcript that corresponds to *NFASC* isoform NF186. To further investigate the disease mechanisms, we reprogrammed fibroblasts from one affected sibling to induced pluripotent stem cells, directed them to neuroepithelial stem cells and finally differentiated to neurons. In early neurogenesis, differentiating cells with selective depletion of the NF186 isoform showed significantly reduced neurite outgrowth as well as fewer emerging neurites. Furthermore, whole-cell patch-clamp recordings of patient-derived neuronal cells revealed a lower threshold for openings, indicating altered Na^+^ channel kinetics, suggesting a lower threshold for openings as compared to neuronal cells without the *NFASC* mutation. Taken together, our results suggest that loss of the full-length *NFASC* isoform NF186 causes perturbed neurogenesis and impaired neuronal biophysical properties resulting in a novel early-onset autosomal recessive ataxia syndrome.

## Introduction

Pediatric neurological diseases are highly heterogeneous in presentation and etiology. Children who present with signs of ataxia; that is, impaired balance and coordination of movement as well as weakness often undergo thorough investigations to determine a specific etiological diagnosis. For those with a progressive disease course and/or a positive family history, the etiology is genetic in a large fraction of the patients (Fogel, 2012). However, despite extensive clinical genetic investigations, not all of these patients receive an etiological diagnosis. This demonstrates the need for further discovery of genes and mechanisms associated with ataxia and other rare neurological disorders.

*NFASC* encodes neurofascin, a cell adhesion molecule belonging to the L1 neural subgroup of the immunoglobulin superfamily (Rathjen et al., 1987; Rathjen and Jessell, 1991) and expressed in a highly complex temporally and spatially regulated pattern. The pre-mRNA undergoes extensive alternative splicing and results in more than 50 different isoforms such as 186, 180, 166, and 155 kDa (Volkmer et al., 1992; Hassel et al., 1997; Kriebel et al., 2012; Suzuki et al., 2017). The isoforms NF155 and NF186 are expressed both in the central nervous system (CNS) and in the peripheral nervous system (PNS) (Nelson and Jenkins, 2017). The glial isoform NF155 is expressed in Schwann cells in the PNS and in oligodendrocytes in the CNS and forms a paranodal axoglial complex with Caspr and contactin, while the cell adhesion molecule, NF186, is expressed in neuronal cells. NF186 has a role in formation of nodes of Ranvier and clustering of Nav channels into the nodes (Nelson and Jenkins, 2017), as well as at the axon initial segments (AISs), where it interacts with ankyrin-G, the master organizer of the AIS, and further recruits and interacts with Nav channels, K channels, and other structural molecules such as βIV spectrins (Zonta et al., 2011; Nelson and Jenkins, 2017). At the AIS, NF186 plays an important role in the maintenance of synapses between inhibitory neurons and the axon. In the cerebellum, these synapses are critical for tuning of the output signal from Purkinje cells, which in turn affects motor coordination (Ango et al., 2004; Kriebel et al., 2012; Desmazieres et al., 2014).

In the present study, we examined two patients, a brother and a sister, with progressive ataxia and muscular weakness. Exome sequencing showed in both patients a novel homozygous mutation (c.3020-1G > A) at the splice acceptor site of intron 25 in the *NFASC* gene, resulting in inframe skipping of exon 26 and absence of the full-length protein isoform NF186. We then used reprogramming and induced pluripotent stem cells (iPSCs) differentiation to generate patient-specific neuroepithelial stem cells (NESCs) and neurons in order to study how the homozygous mutation affects human brain development (Takahashi and Yamanaka, 2006). We show that full-length NF186 is completely lost in patient differentiated cells and that this isoform is important at the early stage of neurogenesis since the mutated patient neural cells sprouted shorter neurites and appeared to be delayed in their neuronal differentiation. In addition, we investigated the functionality of the patient neurons and show deregulated electrophysiology compared to neurons from healthy control individual.

## Materials and Methods

### Human Subjects

The study was approved by the Regional Ethics Committee, Stockholm, and written informed consent was obtained from each participating individual or their respective legal guardians.

### Genetic Investigations

DNA samples derived from blood of affected individuals (III:1 and III:4), the unaffected siblings (III:2 and III:3), and the healthy parents (II:1 and II:2) were enriched with Agilent SureSelect Human All Exon 50M (Agilent), according to manufacturer’s instructions, and were then analyzed by exome sequencing. Library preparation, sequencing, and downstream bioinformatics analyses were performed as previously described (Kvarnung et al., 2018). Confirmation of the candidate variant was done in all members of the family by polymerase chain reaction (PCR) and Sanger sequencing, using standard protocols. Primers are available upon request.

### RNA Analyses in Fibroblasts

Total RNA was extracted from cultured fibroblasts derived from skin biopsies from one affected individual (III:4) and the parents. In a total volume of 20 µl, first-strand cDNA was synthesized with 250 ng of total RNA using random primers and RevertAid First Strand cDNA Synthesis kit (Thermo Scientific). Polymerase chain reaction was performed using primers specific for exons 25 and 27 of *NFASC*. The PCR products were cut out from agarose gel and were purified using GeneJET Gel Extraction Kit (Thermo Scientific). The fragments were sequenced to validate the skipping of exon 26. Isoform specific expression levels of *NFASC* (NF186) were studied by quantitative real-time PCR (qPCR) on Quant-Studio 7 Flex Real-time PCR System (Applied Biosystems) using Power SYBRGreen (Life Technologies). All samples were run in triplicates, averaged, and normalized against total isoforms of *NFASC*. Mathematical model developed by Pfaffl (2001) was used for relative quantification of *NFASC* isoforms.

### Reprogramming Fibroblasts and iPSC Culture in Defined Condition

Skin biopsies were obtained from a patient with *NFASC* mutation (*NFASC c.*3020-1G > A) (III:4), and fibroblast lines were established *via* enzymatic digestion. One unrelated control (Ctrl3) is explained elsewhere (Shahsavani et al., 2017). Fibroblasts at a low passage were used for reprogramming, and iPSC lines were established in xeno-free and defined conditions as previously described (Kele et al., 2016). Briefly, fibroblasts (0.1 × 10^6^ cells) were transduced by introducing four Yamanaka factors: *OCT4*, *SOX2*, *KLF4*, and *C-MYC*, using the nonintegrating *Sendai virus* vectors at a multiplicity of infection of 3 (CytoTune-iPS reprogramming kit; Life Technology) in Essential 8^™^ medium (Gibco) supplemented with 10 ng/ml basic fibroblast growth factor (bFGF). One week late, transduced cells were replated on human recombinant Laminin-521 (Biolamina)–coated plates. Twenty days posttransduction, individual colonies were manually picked, and each colony transferred onto a laminin-coated plate. Induced pluripotent stem cells were passed every 3 to 5 days chemically using TrypLE-Select (ThermoFisher Scientific). Pluripotency of the PSC lines was characterized with the well-established method “PluriTest” as previously described (Shahsavani et al., 2017).

### Generation and Neuronal Differentiation of Neuroepithelial-Like Stem (NESC) Lines

Neuroepithelial stem cells were generated as previously described with some modifications (Shahsavani et al., 2017). Briefly, a confluent homogenous iPSCs were neurally induced by treating with hNoggin (0.5 ng/ml Peprotech), TGFB-inhibitor SB431542 (10 μM; STEMCELL Technologies), and GSK3 inhibitor CHIR (3.3 μM) for 12 days. Noggin and CHIR99021 were maintained throughout the induction and SB431542 only for the first 4 days. Induced cells were plated onto 0.1 mg poly-l-ornithine (Sigma-Aldrich) and 1 μg/ml laminin L2020 (Sigma-Aldrich)–coated plates at high density (40,000 cells/cm^2^) in NESC medium consisting of Dulbecco modified eagle medium/F-12 GlutaMAX supplemented with N2 1%, B27 (0.1%), FGF2 (10 ng/ml), and Pen/Strep all from Invitrogen and EGF (10 ng ml/m1; Peprotech). Established NESC lines were cultured as adherent monolayers, passaged at the ratio of 1:3 every third day with TrypL-Express, and frozen in the NESC medium containing 10% DMSO. FGF2 and EGF were removed from the NESC culture to induce neuronal differentiation. Differentiation cultures were continued for 8 weeks, cells were fed every other day, and cell pellets for RT-qPCR and fixed cells on coverslips for staining were collected every week.

### Immunofluorescence Staining

Immunofluorescent staining was performed according to standard methodology. Briefly, cells were fixed with 4% paraformaldehyde for 20 min; washed with phosphate-buffered saline (PBS); blocked using 5% goat serum, 0.1% bovine serum albumin, and 0.3% Triton-X in PBS; and followed by primary and secondary antibody staining. Antibodies and dilutions are listed in **Supplementary Table 1**.

### Neurite Outgrowth Assay

Neurite outgrowth assay was performed as previously described (Shahsavani et al., 2017).

### Proliferation and Differentiation Assays

Both assays were performed as described previously (Shahsavani et al., 2017). Cells were counted in sextuplicate for the proliferation assay and in triplicate for the differentiation assay using a TC20TM automated cell counter (Bio-Rad). Cell counts were calculated as fold change compared to day 0 of each individual line.

### Western Blot

For Western blot analysis, protein was extracted using Qiagen All-Prep Kit (Qiagen, Hilden, Germany) and manufacturer’s protocol. Protein concentrations were determined by Qubit^™^ Protein Assay Kit (ThermoFisher Scientific) and fluorometer. Samples were run on a 10% sodium dodecyl sulfate–polyacrylamide gel electrophoresis, electrotransferred onto nitrocellulose membrane, immunodetected using the appropriate primary and secondary antibodies, and visualized by LICOR ODYSSEY 9120 imaging system (LI-COR, Cambridge, UK) according to manufacturer’s instructions. Primary antibodies used were Anti-Pan-Neurofascin clone L11A/41 (1:1000; Merck Millipore), mouse monoclonal anti–ankyrin-G (1:1000; Abcam), and mouse monoclonal anti–β-actin (1:1000; Sigma). Secondary antibodies for the LICOR detection system were donkey anti–mouse IgG IRDye 800CW and donkey anti–rabbit IgG IRDye 800CW. Densitometry was performed with Image Studio Lite V 5.2 software.

### Voltage-Clamp Analysis of Neural Cells

Whole-cell voltage-clamp recordings were performed at room temperature (20°C–22°C) after 12 to 16 weeks following the initiation of nondirected NPC differentiation. Axopatch 200B amplifier was used to record currents that were filtered at 2 kHz, digitized online at 10 kHz using an analog-to-digital converter, and then analyzed using pClamp 10.2 software (Molecular Devices, USA) as described (Jin et al., 2011). Recording pipettes were made from borosilicate glass capillaries (Harvard Apparatus Ltd, UK), and pipette resistance was typically 3 to 5 MΩ filled with the intracellular solution. The intracellular solution contained (in mM) 15 NaCl, 135 CsCl, 10 HEPES, and 5 EGTA adjusted to pH 7.3 (all from Sigma-Aldrich) and 289 mOsm. The extracellular solution contained (in mM) 137 NaCl, 4 CsCl, 2 CaCl_2_, 1 MgCl_2_, and 10 HEPES adjusted to pH 7.4 (all from Sigma-Aldrich) and 302 mOsm. A 3M KCl agar bridge was used to minimize the junction potential. The calculated reversal potential for the Na^+^ current is 55 mV using the solutions given above. The experiments were performed under voltage-clamp conditions and currents recorded. The cells were held at a membrane potential of −80 mV, and currents were evoked by voltage steps ranging from −100 to +90 mV in 10-mV increments, and the peak current was measured. Sodium channels were blocked with 1 µM tetrodotoxin citrate (TTX) applied in the extracellular solution. Sodium currents were isolated by subtracting the background currents, measured in the presence of TTX, from initial recordings for each cell measured in the absence of TTX. Data were analyzed with pCLAMP Software version 10.5, Microsoft Excel and GraphPad Prism.

### Statistical Analysis

Statistical tests were performed using IBM SPSS Statistics version 25. Analyses of variance (ANOVAs) were performed if the dataset met the assumptions of a parametric test and were not significant on the Shapiro-Wilk test for normality. If equality of variances was not assumed, a Games-Howell test was used. Data were presented as mean ± standard error of mean, and *p* < 0.05 was considered as statistically significant (*p < 0.05, **p < 0.005, ***p < 0.000).

## Results

### Clinical Findings

**Figure 1:**
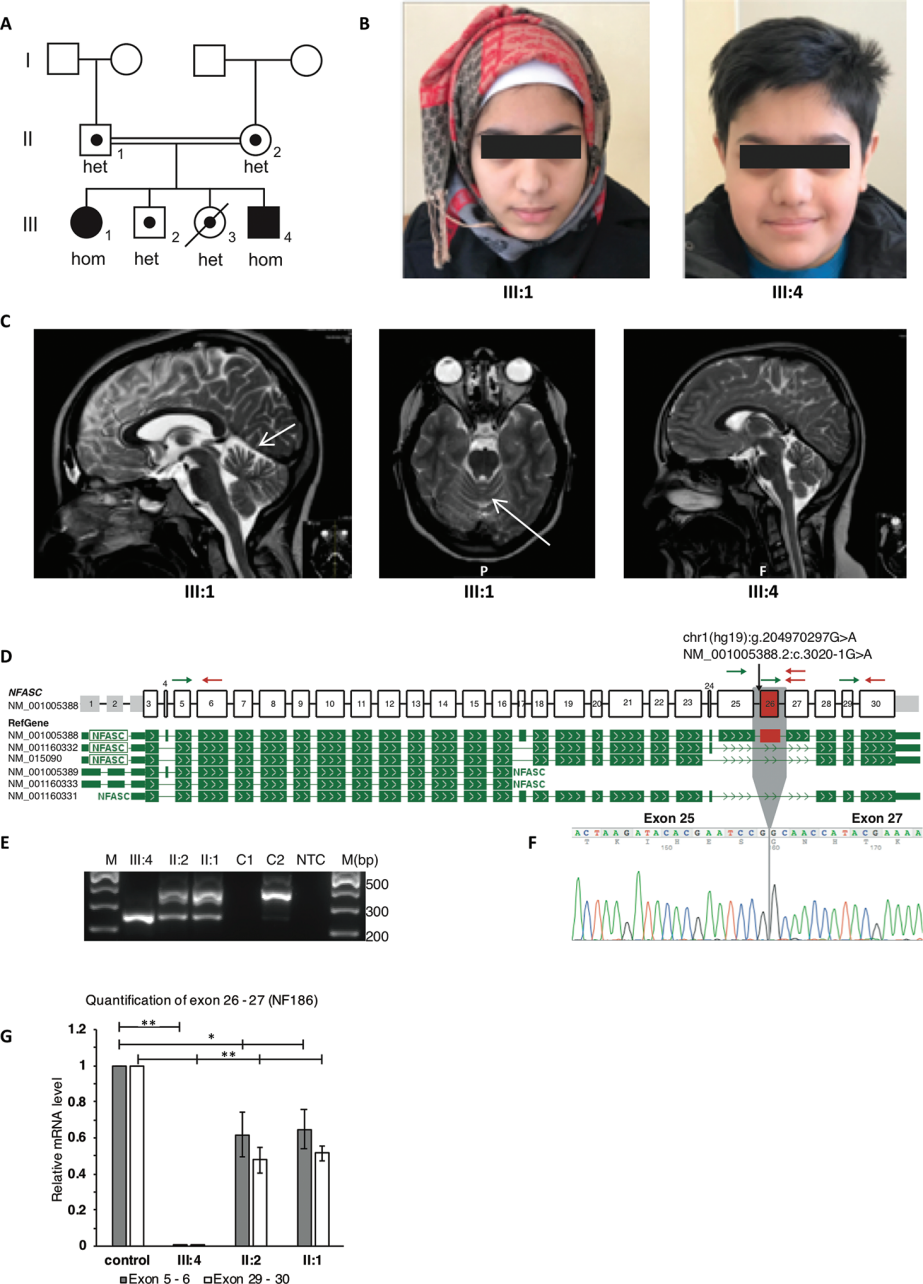
Clinical and molecular features of the patients with homozygous *NFASC* mutations at the splice acceptor of intron 25. The mutation leads to skipping of exon 26 in transcript NM_001005388.2 which encodes for NF186. **(A)** Pedigree of the family. The two affected individuals with a homozygous c.3020-1G > A mutation in *NFASC* are marked with filled symbols. The two unaffected siblings, of whom one has passed away from a severe infection, as well as the healthy parents are marked with dots indicating heterozygous carrier status. Carrier status of family members was identified using both ES and Sanger sequencing. **(B)** Facial photographs of the two affected individuals showing no dysmorphology. **(C)** Magnetic resonance imaging (MRI) of the brain in both patients. T2-weighted scans show in the sister (III:1) mild atrophy of the cerebellar vermis as indicated by arrows on axonal as well as sagittal images. Normal findings were seen in the brother (III:4). **(D)** Schematic representation of *NFASC* exons and RefGene transcript isoforms are shown. Transcript structures are adapted from ProteinPainter application of St. Jude PeCan Data Portal (https://pecan.stjude.cloud/proteinpaint). Black arrow shows the location of the mutation at the splice acceptor site of intron 25 according to transcript NM_001005388.2. Green and red arrows represent forward and reverse primers for reverse transcription PCR and quantitative PCR, respectively. **(E)** Reverse transcription followed by PCR amplification of region between exons 25 and 27 shows a single short fragment (241 bp) in the affected individual compared to the fragment (358 bp) obtained from RNA sample extracted from control fibroblasts C2. The carrier parents have both short and long fragments. RNA extracted from EBV transformed blood cells C1 does not show any NFASC expression. NTC, no template control **(F)** Sanger sequencing of short fragments confirms skipping of exon 26. **(G)** mRNA isolated from fibroblasts that are obtained from skin biopsies of the patient III:4, parents and two controls were used for RT-qPCR. Expression of exon 26 containing transcript isoform NF186 is not detected in patient III:4. The expression level of this isoform in the parents is half compared to the mean expression level of controls when the expression levels normalized to against total *NFASC* isoforms. Significance is indicated as *p < 0.05 and **p < 0.005.

The two affected siblings, an 18-year-old girl and a 13-year-old boy, are the offspring to consanguineous parents originating from Pakistan. A pedigree and facial photographs are shown in **Figures 1A, B**. The affected brother and sister were born at full term after normal pregnancies and with unremarkable neonatal findings. In infancy, the siblings suffered from recurrent upper and lower airway infections with respiratory problems. Motor development was delayed in both siblings with ability to sit at 9 months (brother) and 12 months (sister), walk with support at 18 months (brother) and 30 months (sister), and walk independently at 2 years (brother) and 4 years (sister). For both, gait was unsteady, and there were problems with balance and frequent falls. Language development was within normal limits, but dysarthria has been present from early age. At the age of approximately 5 years, neurologic assessments showed in both siblings ataxia and mild general hypotonia with mild patellar hyperreflexia without other overt pyramidal signs or atrophy. Cognitive level has not been formally assessed in the brother, but appears to be within the normal span. The sister complained about progressive difficulties in school, and psychological testing at the age of 11 years revealed a full-scale IQ of 79 to 86. Neurophysiologic testing (ENeG) of the boy and the girl at the age of 9 and 14 years, respectively, showed normal findings from both upper and lower extremities. Magnetic resonance imaging (MRI) of the brain at the same ages showed mild vermian atrophy in the sister and normal findings in the brother (**Figure 1C**). Visual evoked potentials and electroretinogram showed normal results. Further ophthalmological assessment in the sister at the age of 16 years showed abnormal vertical optokinetic nystagmus (*OKN*) with no response upon stimuli, indicating cerebellar dysfunction. Nystagmus was seen upon fixation, but there was no manifest nystagmus at rest. The clinical course has been marked by progressive motor problems due to a combination of limb weakness (5-/5 both proximally and distally), ataxia, and balance problems. The sister became wheelchair-bound at the age 12 years, and the brother uses a walker indoors and a wheelchair outdoors. In addition, the siblings suffer from disabling vertigo, nausea, and headache.

### Genetic Findings and Effects on Splicing of the *NFASC* Gene

After filtering of variants based on autosomal recessive inheritance model and minor allele frequency below 0.1%, only one single rare variant was detected as candidate. Homozygous variants shared between both affected siblings and heterozygous in the unaffected parents are shown in **Supplementary Table 2**. A homozygous variant c.3020-1G > A at the splice acceptor site of intron 25 in NFASC (NM_001005388.2) was detected in both affected siblings. Segregation analyses showed that the variant was heterozygous in the parents and in the two unaffected siblings. The variant had a CADD (v1.4) score of 34 and was not present in gnomAD (v2.1), and the gene is expressed highly in all the tested brain tissues according to GTEx, which altogether made *NFASC* a very strong candidate gene. The potential effect of the variant on splicing was assessed at RNA level using alternative primer pairs as shown in **Figure 1D** (arrows). The variant leads to an inframe skipping of exon 26 (**Figures 1E, F**). There was no expression of an exon 26–containing isoform in the patient (III:4), and we observed an approximate 50% reduction in the parents when compared to healthy controls (**Figure 1G**).

### Characterization of Patient-Specific and Healthy iPSC and NESC Lines

**Figure 2:**
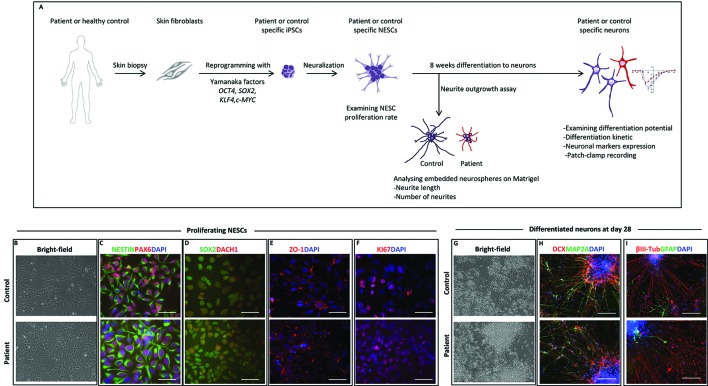
Neural stem cell (NESC) characterization and differentiation. **(A)** Schematic diagram of our *in vitro* model from somatic cells to neurons. **(B)** Bright-field images of iPSC-derived NESCs from control and patient (III:4) in monolayer culture. NESCs self-organized into neural rosette structures. **(C)** Immunofluorescent staining of iPSC-derived NESCs. All derived NESC lines expressed neural stem cell markers NESTIN, PAX6, **(D)** SOX2, DACH1, **(E)** ZO-1, and **(F)** proliferation marker KI67. Nuclei stained with DAPI, scale bar: 50 µm. **(G)** Bright-field images of 28 days’ differentiated neurons of patient and control. **(H)** Immunofluorescent staining of 28 days’ differentiated neurons from patient and control. Differentiated neurons expressed neuronal markers DCX and MAP2A. **(I)** The majority of 28 days’ differentiated cells were positive for the neuronal marker ßIII-tubulin with low amount of cell expressing the glia marker GFAP. Scale bar 100 µm.

To further investigate the role of healthy and mutated NF186 in neural cells, we outlined an *in vitro* disease modeling study using reprogramming and neuronal differentiation of patient-specific cells (**Figure 2A**). We established iPSCs from the affected homozygous brother (III:4) by introducing cDNA of *OCT4*, *SOX2*, *KLF4*, and *c-MYC* into fibroblast cells using the nonintegrating *Sendai virus* vectors. No significant differences were observed at iPSC derivation and characterization, and iPSCs maintained a normal karyotype after reprogramming (**Supplementary Figure 1A**). Induced pluripotent stem cell lines derived from the patient and control were morphologically similarly displaying a large nuclei and small cytoplasm. They were all positive for the nuclei pluripotency markers OCT4 and NANOG (**Supplementary Figures 1B, C**). The iPSC lines passed the PluriTest with a low novelty score demonstrating their pluripotency (**Supplementary Figure 1D**). The exogenous vector expression was confirmed absence by PCR in all lines (data not shown). Then, we used neural induction of the iPSCs to establish NESCs (Falk et al., 2012), a cell type with an equivalent in the developing human hindbrain (Tailor et al., 2013). The iPSC-derived NESCs were stably cultured and robustly expanded in self-organizing neural rosette structures (**Figure 2B**). Immunofluorescent staining showed that NESCs expressed key neural stem cell markers like NESTIN, PAX6, SOX2, DACH-1, and ZO-1, as well as the proliferation marker KI67 (**Figures 2C–F**). Both lines showed a high neurogenic potency, with the majority of the differentiated cells expressing the general neuronal markers βIII-tubulin, doublecortin (DCX), and MAP2A (**Figures 2G, H**). Differentiation of NESCs resulted, as expected, in a high number of neurons and low number of glia cells (**Figure 2I**) (Falk et al., 2012). To further characterize the patient and control neural cells, we investigated the proliferation rate at both the undifferentiated NESC and the neuronal differentiation stages by performing cell counting assays. No differences were observed in proliferation between patient and control lines at the NESC stage with a doubling time of around 24 h (**Supplementary Figure 1E**). During differentiation, when growth factors were removed, proliferation naturally slowed down, and neural stem cells leave the cell cycle and differentiate into postmitotic neurons, at the inspected stages of differentiation we did not observe any significant differences in proliferation kinetics among lines (**Supplementary Figure 1F**).

### *NF186* Expression During Early Human Neuronal Differentiation

**Figure 3:**
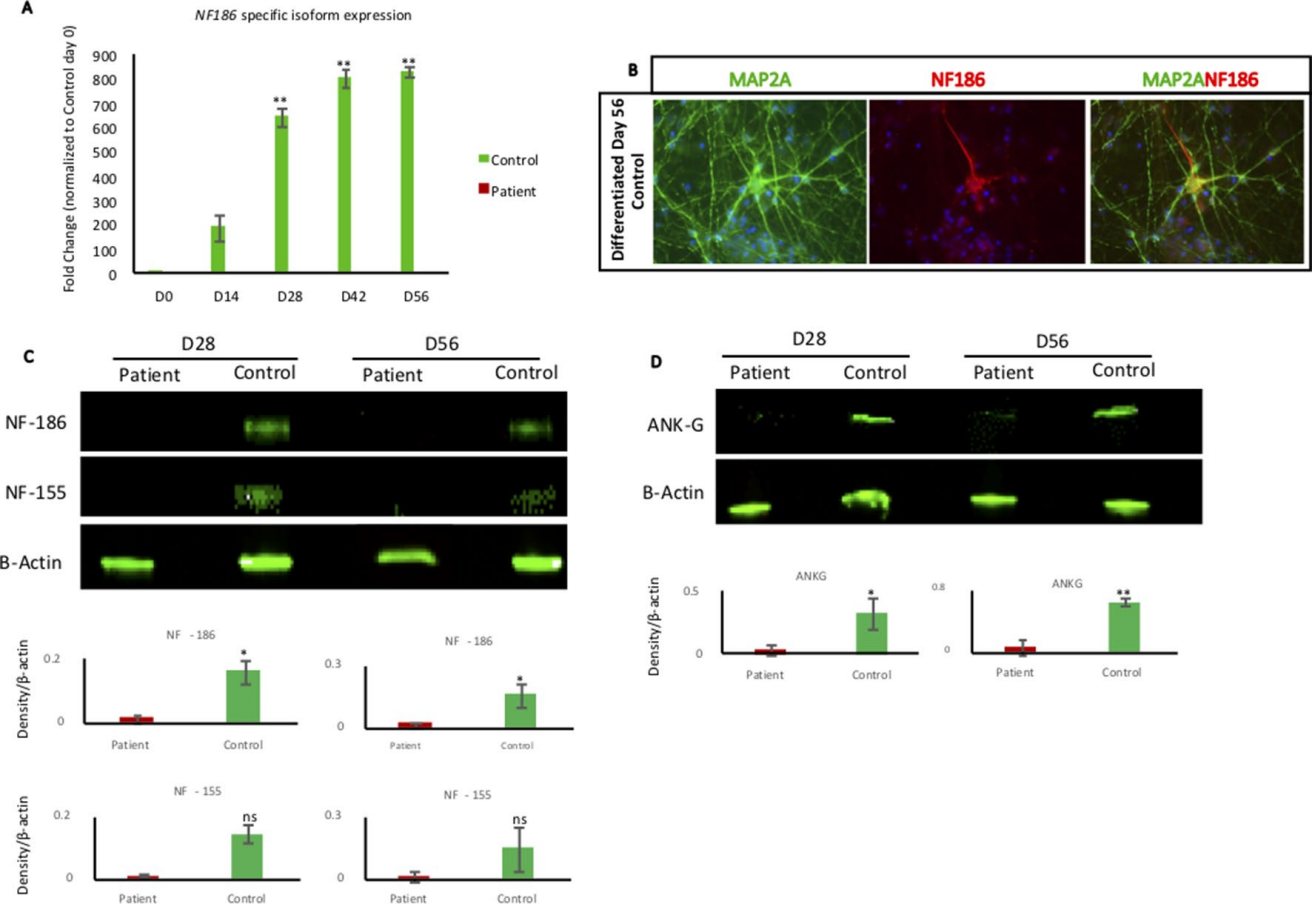
NF186 isoform expression during neural differentiation. **(A)** RT-qPCR analysis of *NF186* isoform using NESCs (day 0) and collected cells at different time points of differentiation (days 14, 28, 42, and 56) from patient and control. Control cells upregulated *NF186* mRNA soon after growth factors removal, while patient cells did not express any *NF186* mRNA containing exon 26. Results represented as fold change. Relative expression values were determined after normalizing the mRNA levels to GAPDH and to day 0 of Control. n = 3 biological replicates per cell line per time point. Error bars represented ± standard error of mean; Significance is indicated as *p < 0.05, **p < 0.005. Primers are listed in **Supplementary Table 3**. **(B)** Immunofluorescent staining of NF186 and MAP2A in 56-day-old neuronal differentiation culture of control cells. NF186 expression was excluded from the dendrite marker MAP2A. Scale bar 50 µm. **(C)** Western blot indicating the expression of NF186 and NF155 in patient and control cells at days 28 and 56 of neuronal differentiation. Densitometry analysis showed significant reduction of NF186 in patient cells, while NF155 reduction was not significant at both days of analysis. Protein expression was normalized to expression of β-actin. **(D)** Western blot showing expression of ankyrin-G in patient and control cells at days 28 and 56 of differentiation. Densitometry analysis showed significant reduction of ankyrin-G in patient cells at day 28 (*p* ≤ 0.05) and day 56 (*p* ≤ 0.001). Protein expression was normalized to expression of β-actin. n = 3 biological replicates per cell line per time point. Error bars represented ± standard error of mean; Significance is indicated as *p < 0.05, **p < 0.001, ns, not significant.

Next, we wanted to examine the expression of the NF186 isoform in our neural cell model during a time course of 8 weeks’ differentiation toward neurons. Quantitative PCR analysis showed that the *NF186*-specific isoform was robustly and significantly upregulated when control NESCs differentiated, while the patient’s cells neither expressed nor upregulated *NF186* (**Figure 3A**). The expression of *NF186* in control cells was upregulated over 500-fold during the early phase (first 4 weeks) of NESC differentiation indicating an important role for NF186 during human neurogenesis (**Figure 3A**). We could also detect expression of NF186 by immunostaining with an NF186-specific antibody at day 56 of differentiation; NF186 expression was excluded from the dendritic marker MAP2A in the differentiating culture of control neurons (**Figure 3B**). Western blot analysis with a pan-antibody specific for NF186 and NF155 showed significant (*p* ≤ 0.05) difference in expression of NF186 between patients and control neurons at both days 28 and 56 of differentiation (**Figure 3C**). There was a trend in higher expression of NF155 in control neurons than in patient neurons at days 28 and 56 of differentiation, but this was not statistically significant (**Figure 3C**). In addition, we detected a significant reduction of ankyrin-G in patient neurons compared to control neurons at day 28 (*p* ≤ 0.05) and day 56 (*p* ≤ 0.001) of differentiation (**Figure 3D**). Taken together, the Western blot analysis validated the qPCR data of the absence of full-length NF186 protein during neurogenesis in our cellular model. In addition, the expression of ankyrin-G was significantly reduced in patient cells lacking full-length NF186.

### NFASC Mutant Cells Displayed Aberrant Neurogenesis and Neurite Outgrowth

**Figure 4:**
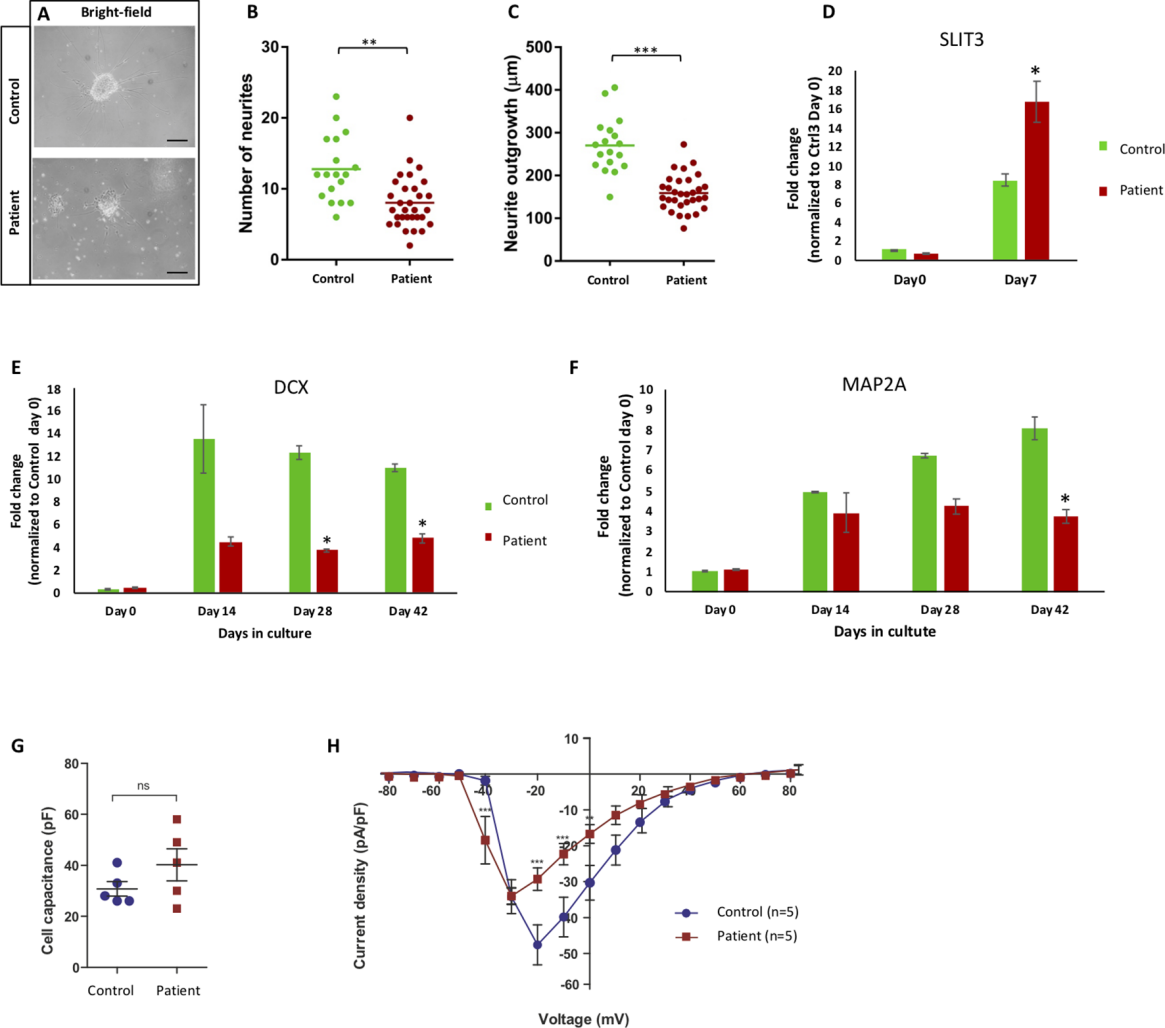
Aberrant neurite extension and different sodium channel recording. **(A)** Bright-filed microscopy of control and patient Matrigel-embedded neurospheres. **(B**, **C)** Graphs from quantified data analysis representing number and length of neurites from control and patient neurospheres. **(B)** Number of emerging neurites from patient neurospheres was significantly fewer than control. **(C)** Average length of patient neurites was significantly shorter that control neurospheres. Each dot represents the average number of neurites or neurite length per neurosphere, and the vertical line represents the mean per cell line. Significance is indicated as *p < 0.05; **p < 0.005; ***p < 0.000. **(D)** mRNA expression of *SLIT3* during differentiation in NESC (day 0) and day 7 from patient (III:4) and control lines. Patient cells significantly upregulated *SLIT3* mRNA upon 1-week differentiation. **(E**, **F)** mRNA expression of neuronal differentiation markers *DCX* and *MAP2A* at NESC (day 0) and differentiation time points (days 14, 28, and 42) in control and patient cell lines. Patient cells expressed less *MAP2A* and *DCX* compared to control upon differentiation. **(G)** Capacitance measurements to compare cell sizes between iPSC-derived neurons from control (Ctrl, blue circles) and patient (red squares). Unpaired *t* test with Welch’s correction (control n = 5, patient n = 5) indicates similar capacitance for neurons from both lineages. **(H)** Current-voltage relationship of whole-cell currents of iPSC-derived neurons from control (Ctrl) and patient. Data points represent average sodium current density [I_Na_ (pA)/size of the cell (pF)] as a function of the membrane potential (mV). The current density in cells derived from patient is reduced when compared to those of control (**p < 0.001; ***p < 0.000). In addition, Na^+^ current is activated at a 10-mV more negative membrane potential in neurons derived from patient when compared to those from control. Statistical analysis was performed by two-way ANOVA following Bonferroni posttest (Ctrl3 n = 5, III:4 n = 5). All errors: ± standard error of mean.

To investigate if the lack of full-length NF186 protein in patient neurons would cause deregulated neurogenesis, we derived neurospheres and studied the neurite outgrowth during the differentiation of the patient and control cells. Both cell lines formed neurospheres, and neurites could be detected both by morphological inspections (**Figure 4A**). However, when quantifying the number and length of the neurites, we found both fewer and shorter neurites of the patient neurons in comparison to control neurons (**Figures 4B, C**). The average number of emerging neurites from the patient and control neurospheres was 8 and 12, respectively (**Figure 4B**). Moreover, the mean length of patient neurites (123 µm) was significantly shorter and less than half the length compared to control neurites (270 µm) (**Figure 4C**). We have previously reported that overexpression of the repulsive axon guidance ligand SLIT3 (Brose and Tessier-Lavigne, 2000) causes shorter neurites (Shahsavani et al., 2017). Indeed, we could detect a significant overexpression of SLIT3 in patient cells during neuronal differentiation, indicating a possible mechanism for the aberrant neurite outgrowth (**Figure 4D**). The kinetic of the neuronal differentiation process is well characterized in our cellular model, and typically, it follows the same timeline in cell lines from healthy individuals (Lundin et al., 2018). Here, we found that differentiating cultures of patient cells did not upregulate neither the immature or the more mature neuronal differentiation markers DCX and MAP2, respectively, as much as healthy control cells (**Figures 4E, F**). Taken together, neurons lacking the full-length NF186 isoform display aberrant neurogenesis *in vitro* with shorter and fewer neurites in addition to less upregulation of neuronal differentiation markers.

### Altered Electrophysiology of Patient Neurons

We have shown that the neurogenesis in our cellular model is deregulated in patient-specific cells lacking the full-length NF186 protein. To investigate how the atypical neurogenesis would influence the function of the neurons, we performed whole-cell patch-clamp recordings in 3 months or older neurons. The cells were held at a membrane potential of −80 mV, and inward sodium currents in response to depolarized membrane potentials were recorded in neurons derived from the patient and a healthy control donor. The neurons from both lineages were similar in size as shown by capacitance measurements (**Figure 4G**). In order to isolate the sodium current associated with opening of sodium channels, we subtracted the nonspecific leakage currents. This revealed that the Na^+^ currents in patient-derived neurons were activated at about 10-mV more negative membrane potentials than the Na^+^ channels in the control neurons (**Figure 4H**). Furthermore, the peak-current amplitude for current activation was lower in the patient-derived iPSC neurons. Since the cells are of similar size, the results indicate that either fewer Na^+^ channels are expressed in the cells from the patient or that the channel kinetics is altered. Furthermore, since the Na^+^ channels in the cells from the patient open at lower membrane potentials, they need smaller membrane depolarizations to open and, thereby, have lower threshold for openings. Thus, the function of patient’s neurons deficient of the full-length NF186 isoform is different compared to neurons from a healthy control individual.

## Discussion

Here, we report two siblings with homozygous NFASC mutations resulting in specific loss of the full-length of isoform NF186. The clinical presentation is distinct, with symptoms that we interpreted as cerebellar (ataxia, dysarthria, poor balance, and vertigo) as the main findings. In addition, the phenotype includes muscle weakness, which appears to be mainly of central origin, and mild cognitive impairment. There are previous publications with convincing indications that loss of neurofascin in general, and NF186 in particular, causes cerebellar dysfunction and ataxia in animals (Zhou et al., 1998; Ango et al., 2004; Zonta et al., 2011; Buttermore et al., 2012; Leterrier et al., 2017). A study of rat hippocampal and cortical organotypic slices found that shRNA knockdown of nf186 caused a progressive AIS disassembly and reduction of ankyrin-G expression (Leterrier et al., 2017).

By using mice deficient for the cerebellar isoform of ankyrin-G, Zhou et al. (1998) and Ango et al. (2004) showed that NF186 and other axonal components of ankyrin-G had impaired localization to AISs of Purkinje cell neurons, resulting in reduced pinceau synapse formation. Mutant mice in the study from Zhou et al. (1998) exhibited a progressive ataxia beginning around postnatal day P16 and subsequent loss of Purkinje neurons with the cerebella of adult mutant mice (>5 months old) being substantially smaller than those of control littermates (Zhou et al., 1998; Ango et al., 2004). Further, Buttermore et al. (2012) showed that conditional knockout of neurofascin in adult Purkinje neurons of conditional knockout mice prevented maturation of the AIS and resulted in loss of Purkinje neuron spontaneous activity, which led to progressive ataxia and neurodegeneration in mice. Thus, the main clinical finding of ataxia in our patients is supported by reports from animal studies.

Muscle weakness and cognitive impairment, which were subtle findings in our patients, have not been specifically studied in neurofascin knockdown animal models. However, neurofascin seems to be involved in learning as suggested by Zitman et al. (2016), who showed that neurofascin knockdown in the hippocampus of rats resulted in impaired learning under stress.

In the literature, there are a few reports of patients with biallelic *NFASC* mutations. One single case, reported by Smigiel et al. (2018), harbored a homozygous nonsense mutation and presented with neonatal severe peripheral hypotonia. This mutation caused selective loss of the NF155 isoform, and as opposed to our cases, NF186 was not affected (Smigiel et al., 2018). A clinical diagnosis of epilepsy and no pupillary response to light indicated affection of the CNS, but the etiology may be difficult to establish since the patient also suffered from central hypoxia with ischemic signs on MRI of the brain. Our patients represent the counterpart to the patient in the study of Smigiel et al. (2018). It is interesting that our patients with selective loss of full-length NF186 exhibit neurological signs and symptoms primarily of central origin, while the PNS currently appears unaffected according to the nerve conduction testing at the time of test performed. However, we cannot exclude subclinical affection of the PNS or the emergence of peripheral symptoms with time. A second report of two siblings with a homozygous missense mutation in *NFASC* was recently published. The phenotype of those patients also included ataxia, in line with the conclusions in our study (Monfrini et al., 2019).

To investigate the underlying cause of symptoms in the patients, we took advantage of the reprogramming technique to derive a cellular model using patient-specific iPSCs and neuronal differentiation. Using the model, we could study the function of the NFASC isoform NF186 in early human neurogenesis since the neural cells of the patient lacked *NF186* mRNA and full-length protein expression. We show that the patient neural stem cells could generate neurons, but the differentiated bulk culture expressed lower levels of the neuronal markers DCX and MAP2 compared to control cultures of neurons indicating a delay in differentiation and maturation. Reports have shown that absence of NF186 reduces the expression of ankyrin-G (Zonta et al., 2011; Alpizar et al., 2019), encouraging us to investigated the protein expression of ankyrin-G in patient and control cells on days 28 and 56 of differentiation. In line with previous publication, our data showed significant reduction of ankyrin-G in patient cells. As full-length NF186 and ankyrin-G play a central role in the AIS organization (Alpizar et al., 2019) and based on our results that both of them were significantly reduced in patient cells, we hypothesized that the emergence of neurite might be affected in the patient neurons. Subsequently, we investigated the neurites of the patient neurons finding that the neurites were significantly fewer and shorter compared to healthy control neurons. The known role of NFACS in AIS, for establishing and maintaining neuronal connectivity, prompted us to investigate the electrophysiological properties of differentiated patient-derived neurons. When inward sodium currents were recorded in neurons differentiated for 3 months, we observed that neurons lacking NF186 were activated at about 10-mV more negative membrane potentials and showed a lower sodium peak-current amplitude than those recorded in control neurons of similar size. The results indicate altered channel kinetics and possibly a decreased number of channels in the absence of NF186. This suggests that the patient-derived neurons have a lower threshold for channel openings and excitation. The abnormalities in electrophysiological properties and neurite formation in our patient-derived neural cells devoid of full-length NF186 suggest detrimental effects on normal brain development. Patient cells will thus fail to extend neurites properly resulting in perturbed coordination and crosstalk with the neighboring cells. This is in line with reports from animal models showing altered action potentials in neurons with a conditional loss of neurofascin, specifically in Purkinje cells (Zonta et al., 2011; Buttermore et al., 2012).

## Conclusion

In aggregate, our results strongly suggest that loss of the full-length NF186 isoform of neurofascin causes an early-onset progressive ataxia syndrome. Functional *in vitro* studies utilizing patient-derived neural cells harboring a homozygous NFASC mutation showed a complete loss of the full-length NF186 protein and that both neuronal differentiation and function were affected, illustrated by fewer and shorter neurites as well as a direct effect on the Na^+^ channels. We therefore propose *NFASC* as a novel autosomal recessive disease gene associated with a phenotype that includes progressive cerebellar symptoms (vertigo, ataxia, problems with balance and motor coordination), muscle weakness, and mild cognitive impairment. The study sheds light on the growing number of isoform-specific disorders and illustrates the importance of human neural *in vitro* models in order to identify mechanisms behind neurological disorders that may serve as platforms for the development of future treatment options.

## Data Availability

The raw data supporting the conclusions of this manuscript will be made available by the authors, without undue reservation, to any qualified researcher.

## Ethics Statement

The studies involving human participants were reviewed and approved by the Regional Ethics Committee, Stockholm. Written informed consent to participate in this study was provided by the participants’ legal guardian/next of kin. Written informed consent was obtained from the individual(s), and minor(s)’ legal guardian/next of kin, for the publication of any potentially identifiable images or data included in this article.

## Author Contributions

MK, MN, and AF conceived the study. MK, AN, and B-MA collected clinical information. FT and DN conducted the bioinformatics analysis. MK, AgL, and FT performed genetic experiments. MS, MM, NB, LL, JS, and ZJ performed stem cell experiments. AF, MS, MM, ND, and BB analyzed the cellular data. MK, MS, FT, MM, ND, BB, AL, and AF drafted the manuscript. AF, EL, and AL supervised the entire study. All the authors approved the final version of the manuscript.

## Funding

Financial support was provided by grants from the Swedish Medical Research Council and the Stockholm County Council (MK, AL). Grant from Swedish Research Council [2015-02424 (ND), 2017-03407 (AF), 2017-02936 (AL)], Stiftelsen för strategisk forskning, SSF [IB13-0074 (AF)], Hjärnfonden (ND, AF, AL, AN), Stiftelsen Sävstaholm (LL), and Bertil Hållsten Research Foundation (AN).

## Conflict of Interest Statement

The authors declare that the research was conducted in the absence of any commercial or financial relationships that could be construed as a potential conflict of interest.
