# Supplementary figures and images for: Ataxia in Patients With Bi-Allelic NFASC Mutations and Absence of Full-Length NF186
Source: Front Genet. 2019 Sep 24;10:896. doi: 10.3389/fgene.2019.00896 (PMC6769111; doi:10.3389/fgene.2019.00896)

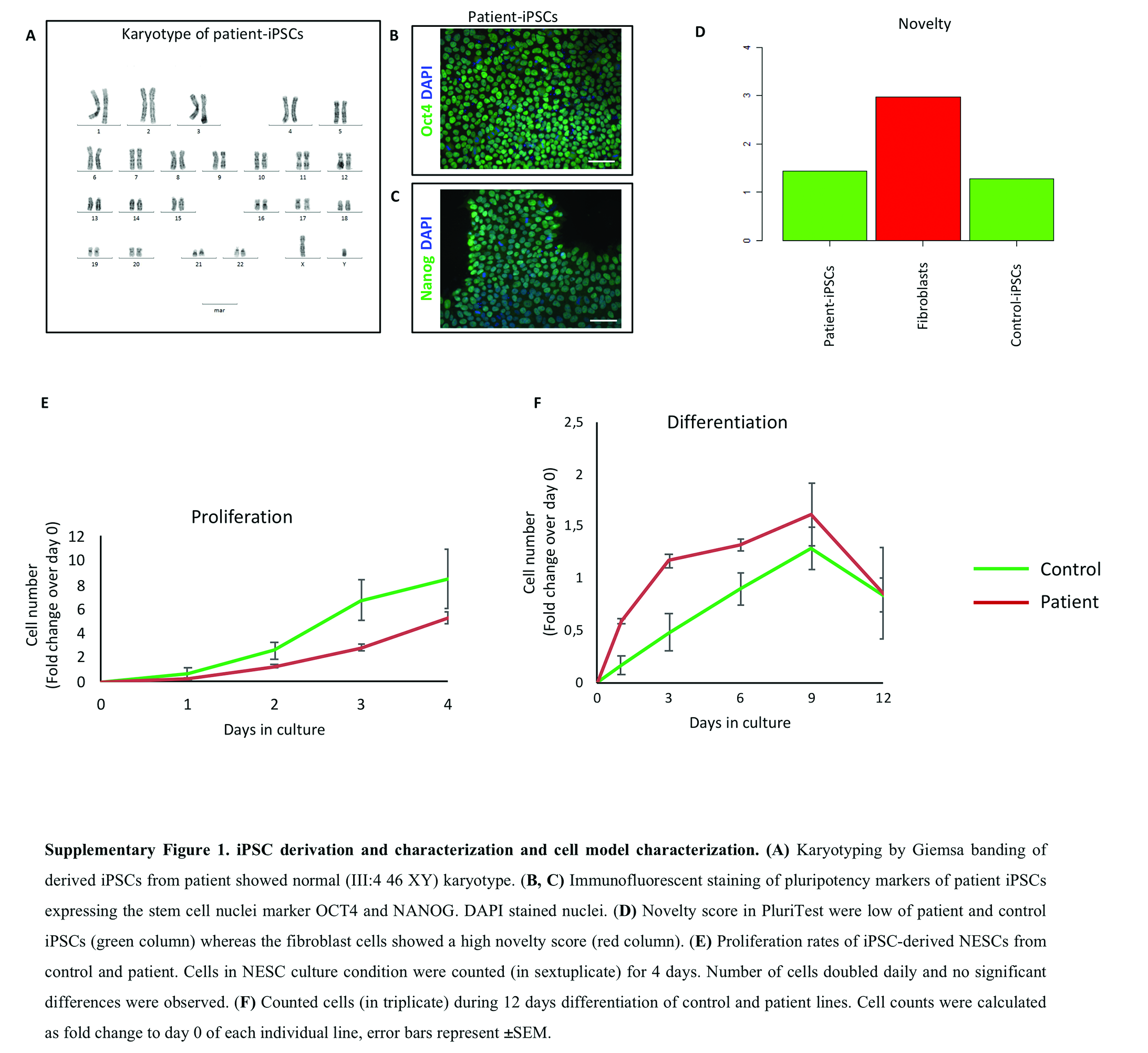

Supplement: Supplementary file 1 [file Image_1.tif]
