# Supplementary material for: Ataxia in Patients With Bi-Allelic NFASC Mutations and Absence of Full-Length NF186
Source: Front Genet. 2019 Sep 24;10:896. doi: 10.3389/fgene.2019.00896 (PMC6769111; doi:10.3389/fgene.2019.00896)
Supplement: Supplementary file 2 [file Table_1.docx]

| Antibody | Dilution | Vendor |
| --- | --- | --- |
| AnkG, m | 1:300 | Abcam |
| Anti-Pan-Neurofascin, m | 1:1000 | Millipore |
| β-actin, m | 1:1000 | Sigma |
| anti–mouse IgG IRDye 800CW | 1:1000 | LI-COR |
| anti–rabbit IgG IRDye 800CW | 1:1000 | LI-COR |
| DACH1, rb | 1:100 | Proteintech |
| DCX, rb | 1:500 | Cell signalling |
| KI67, rb | 1:250 | Abcam |
| MAP2A, m | 1:500 | Abcam |
| NESTIN, m | 1:200 | Millipore |
| NF186, rb | 1:1500 | Cell signalling |
| PAX6, rb | 1:500 | Biolegend |
| SOX2, m | 1:200 | Millipore |
| ßIII-Tubulin, rb | 1:1000 | Biolegend |
| ZO-1, rb | 1:100 | Invitrogen |

Supplementary Table 1. List of antibodies used for immunocytochemical stainings. rb: Rabitt, m: mouse).
