# Supplementary material for: Ataxia in Patients With Bi-Allelic NFASC Mutations and Absence of Full-Length NF186
Source: Front Genet. 2019 Sep 24;10:896. doi: 10.3389/fgene.2019.00896 (PMC6769111; doi:10.3389/fgene.2019.00896)
Supplement: Supplementary file 4 [file Table_3.docx]

| Primers | Forward (5’-3’) | Reverse (5’-3’) |
| --- | --- | --- |
| NF186 | TACTACAACCACTGCTGCCG | TGCTCATCAGGGGCGGATT |
| SLIT3 | AACTCCATCAAAGCCATCC | GGCATCTGGAGCAATATCC |
| MAP2A | GCCAATGGATTCCCATACAG | TGGAGAAGGAGG |
| DCX | GCAGTCTCCCATCTCTACGC | ATGGAATCACCAAGCGAGTC |
| GAPDH | CAGATTAGCTCTGCTCCTGTTCGAC | ACGACCAAATCCGTTGACTC |

Supplementary Tabel 3. Primers sequences
